# Chiral Linked Systems as a Model for Understanding D-Amino Acids Influence on the Structure and Properties of Amyloid Peptides

**DOI:** 10.3390/ijms23063060

**Published:** 2022-03-11

**Authors:** Aleksandra A. Ageeva, Alexander B. Doktorov, Nikolay E. Polyakov, Tatyana V. Leshina

**Affiliations:** 1 Voevodsky Institute of Chemical Kinetics and Combustion, 630090 Novosibirsk, Russia; al.ageeva@gmail.com (A.A.A.); polyakov@kinetics.nsc.ru (N.E.P.); leshina@ngs.ru (T.V.L.); 2 Department of Natural Sciences, Novosibirsk State University, 630090 Novosibirsk, Russia

**Keywords:** chiral linked systems, diastereomers, D-amino acids, amyloid peptides, electron transfer, chiral inversion, spin effects, fluorescence quenching, molecular dynamics

## Abstract

In this review, we provide an illustration of the idea discussed in the literature of using model compounds to study the effect of substitution of L- for D-amino acid residues in amyloid peptides. The need for modeling is due to the inability to study highly disordered peptides by traditional methods (high-field NMR, X-ray). At the same time, the appearance of such peptides, where L-amino acids are partially replaced by D-analogs is one of the main causes of Alzheimer’s disease. The review presents examples of the use diastereomers with L-/D-tryptophan in model process—photoinduced electron transfer (ET) for studying differences in reactivity and structure of systems with L- and D-optical isomers. The combined application of spin effects, including those calculated using the original theory, fluorescence techniques and molecular modeling has demonstrated a real difference in the structure and efficiency of ET in diastereomers with L-/D-tryptophan residues. In addition, the review compared the factors governing chiral inversion in model metallopeptides and Aβ42 amyloid.

## 1. Introduction

**Figure 1:**
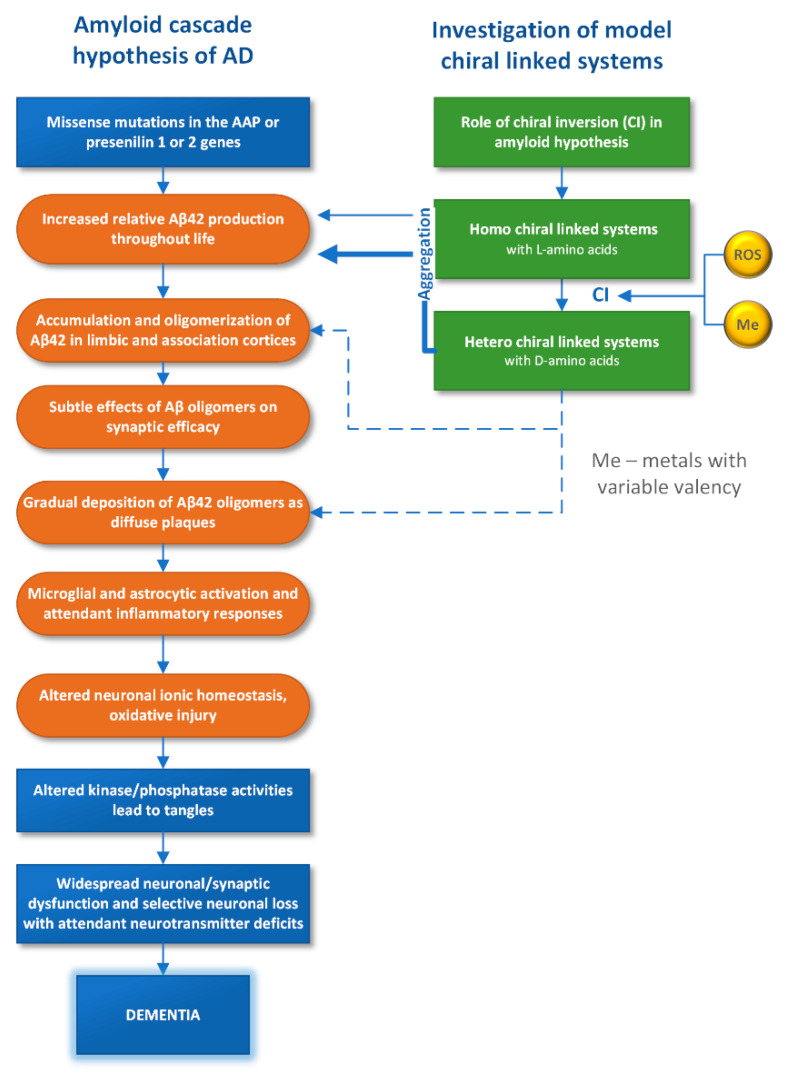
The amyloid cascade hypothesis for Alzheimer’s disease and results of the model systems investigation that provides insight into the role of D-amino acid participation in the amyloid Aβ42 transformation.

Today, the properties of D- and L-optical isomers of amino acid residues in proteins and peptides are in the focus of interests of scientists in various specialties. The source of this interest is associated with the fact that one of the main causes of neurodegenerative diseases (Alzheimer’s, Parkinson’s, and some others) is due to the replacement in certain proteins and peptides of L-amino acids to D-analogs, which occurs during aging [1,2]. It is these proteins and peptides are believed to undergo disruptions in folding processes, such as aggregation resulted in the formation of large oligomers and fibrils that are highly toxic, especially, for the brain cells [3,4]. So, β-amyloid (Aβ42), a 39–42 amino acid polypeptide found in the brain, being present in a high concentration in the central nervous system, penetrates the membranes of astrocytes, forming special channels through which it affects the level of extracellular calcium [5,6]. Aβ42 also changes the mitochondrial potential of cells (astrocytes and microglia), produces toxic ROS, and indirectly, through intercellular exchange with astrocytes, causes neuronal death [6,7,8]. For example, the so-called “amyloid” hypothesis of the origin of the disease associated with the complete destruction of the psyche—dementia, widely represented in the literature, is shown in Figure 1. The processes mentioned above with the participation of Aβ42 are marked in the diagram by orange color.

**Figure 2:**
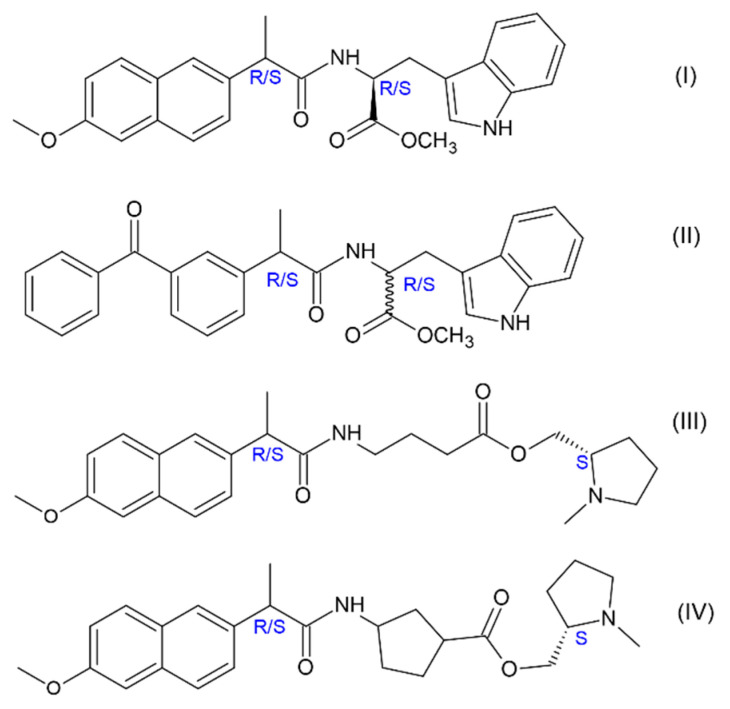
Structures of the model linked systems—dyads (**I**–**IV**).

The mechanisms of the Aβ42 monomer action and its aggregated forms have been studied in cell models, while the primary cause of the formation of aggregates and their relationship with the inclusion of D-amino acids in biomolecules remains to be studied [3,4,9,10,11]. This review describes first results on the influence of D-amino acids on the structure and properties, obtained in the study of model systems (Figure 1). Since such methods as high-resolution NMR and X-ray cannot be applied for investigation of highly disordered proteins and peptides, the use of model systems—short peptides, studied by methods in silico is considered as a trend [3]. This review discusses works that implement a modification of this approach. The modified approach associated with the use of elementary photoinduced processes is described in detail in the works [12,13,14]. New approach employs model linked systems—donor-acceptor dyads, with L- or D-residues of tryptophan (Trp), which allow one to use spin effects and fluorescence quenching to study the difference in the reactivity of optical isomers in the intramolecular photoinduced electron or hydrogen atom transfer [15,16,17]. The results are compared with molecular modeling data [17]. The quenching of D- and L-Trp excited state occurs in the dyads, where acceptors are the well-known non-steroidal anti-inflammatory drugs (NSAIDs): (*R/S*)-naproxen (NPX) and (*R/S*)-ketoprofen (KP) (Figure 2). The choice of NSAIDs is not accidental, since the derivatives of these drugs are now being tested as inhibitor of γ-secretase—a complex of enzymes involved in the synthesis of Aβ42 from precursor of amyloid protein (APP) [18,19].

Methods of the exploration of model systems—dyads are spin chemistry (chemically induced dynamic polarization of nuclei (CIDNP) and electrons (CIDEP)) and optical spectroscopy (quenching of the fluorescence). The CIDNP and CIDEP phenomena are described everywhere, see for example [14,15,16]. Analysis of CIDNP and CIDEP effects demonstrated significant differences in the magnetic resonance characteristics of short-lived paramagnetic forms—biradical-zwitterions (BZ) of (*S*,*S*)-, (*R*,*S*)- and (*S*,*R*)-dyads, while the fluorescence data showed differences in their quenching (stereodifferentiation) [12,13,14,15,16,20]. Molecular dynamics (MD) simulations, in turn, showed differences in the conformations of dyads with different optical configurations [20]. Spin selectivity (SS), a new property of chiral systems recently discovered by the examples of dyads with NPX (I, III-IV), i.e., the difference in the CIDNP enhancement coefficients of diastereomers, was also characteristic for diastereomers of dyad II [20]. However, in contrast to the previously studied dyads with NPX, where the donors are (*S*)-N-methylpyrrolidine and L- and D-Trp (I, III-IV), the **SS** values for dyad II diastereomers are larger by an order of magnitude. This difference was assumed to be related to the association of diastereomers. Actually, the dependences of the **SS** on the ratio of diastereomers concentrations in solutions under the UV irradiation of their mixture turned out to be different for dyads with NPX and KP [20]. So, it is most likely that the sizes of associates formed from these dyads are also different. This is the reason of an order of magnitude difference in the spin selectivity for dyads I and II, because the modified CIDNP theory showed that the size of particles (associates) can strongly influence on the magnitude of hyperpolarization. This effect was accounted for the suppression of the singlet-triplet conversion in the BZ under the action of the dipole-dipole interaction of electrons [20].

In the review, considerable attention is paid to **SS**, reflecting the difference in the spin density distribution that points to the difference in the electron density distribution. The latter may be one of the real reasons for the differences in the biological activity of both the enantiomers of drugs and amino acids. Analysis of CIDNP effects and fluorescence quenching data for (*S*,*S*)-, (*S*,*R*)- and (*R*,*S*)-diastereomers of dyad II allowed the authors to conclude that dyads with D- and L-Trp really show different properties: stereodifferentiation, **SS** and inclination to the association. The main goal of this review is to show that model systems can shed light on some significant problems for understanding the nature of the neurodegenerative diseases: the actual mechanism of chiral inversion (transformation of L-amino acids to D-analogs) and differences in the behavior of systems with L- and D-optical isomers of amino acids [1]. Furthermore, many sources point to the toxic effects of free radicals and reactive oxygen species on the progression of neurodegenerative ailments [5,6,7,8,9]. The review presents a section describing the factors affecting chiral inversion in biological and model systems, as well as the evidence for the participation of radical species and metals with variable valence in this process. Thus, the authors of this review hope to demonstrate the fruitfulness of the chosen approach, since the experimental study of model systems in combination with MD helps to take a step towards understanding of the elementary mechanisms of the processes occurring in proteins and peptides in living systems.

## 2. Reactivity of Optical Isomers of Dyads I and II According to the CIDNP Data and Fluorescence Quenching

In this section, we discuss the differences in the efficiency of elementary chemical reaction—single-electron transfer (ET) and energy transfer in different optical configurations of the simplest linked systems—donor-acceptor dyads. Dyads, where one partner is a chiral NSAID and the other is a chiral amino acid residue, have gained popularity as the simplest models of chiral drug interactions with chiral amino acid residues located in the active site of enzymes, including COX 1,2 [12,13]. It is the interaction of two chiral particles in the active site of the enzyme and receptors that can explain the differences in the therapeutic activity of drugs enantiomers, in particular, NSAIDs. Indeed, although enantiomers are known to have identical physicochemical properties, the diastereomer analog formed in the active site already has physical prerequisites for differences in the reactivity of the partners. Photochemistry and spin chemistry studies allowed authors to trace the differences between the (*R*)- and (*S)*-enantiomers of NSAIDs that are part of the diastereomers [12,13,14]. The differences in the ET efficiency have been described in detail earlier, and there is some correlation with the difference in the medicinal properties of naproxen enantiomers [12,13]. Note, that knowledge of the elementary stages underlying these differences is a challenging problem of pharmacology and medicinal chemistry [17]. Further, the difference in the reactivity of L- and D-amino acid residues in dyads with NPX and KP (I, II) will be described.

**Scheme 1:**
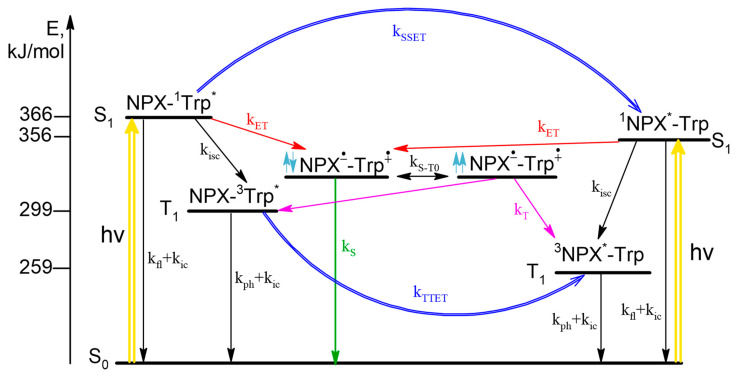
Photoinduced processes in dyad I. Subscripts denote fl—fluorescence, ic—internal conversion, isc—intersystem crossing, ph—phosphorescence, exc—exciplex, S-T_0_—intersystem crossing in BZ, *—excited states.

According to the CIDNP and fluorescence data, the scheme of photoinduced processes in dyad I is as follows (Scheme 1). Upon photoexcitation the singlet excited state of chromophore is formed, then ET may occur both from Trp in ground state to ^1^NPX* in singlet excited state and from singlet excited state of ^1^Trp* to NPX in ground state resulted in formation of BZ. The fraction of light absorbed by amino acid and drug molecules depends on excitation wavelength. In CIDNP experiments λ_exc_ = 308 nm and all light is absorbed by NPX (97%), at λ_exc_ = 300 nm in fluorescence experiments 40% of light is absorbed by Trp and 60% by NPX. From experimental data, the rate of back ET from triplet spin state (k_T_) of BZ was established to be higher than from singlet (k_S_). In addition, the singlet ^1^Trp* and triplet ^3^Trp*excited states are quenched via singlet-singlet (SSET) and triplet-triplet energy transfer (TTET), correspondingly. In essence, photoinduced processes related with CIDNP formation are limited by the right part of the scheme that describes ^1^NPX* quenching (Scheme 1).

In the case of the dyad II, according to the literature data, the singlet excited state of ^1^Trp* can be quenched by both ET and SSET from Trp to KP. The stages with ET in diastereomers of the dyad were studied using CIDNP technique. An extremely low proton polarization of the (*R*,*S*)/(*S*,*R*)-diastereomer compared to (*S*,*S*) was found. CIDNP analysis shows that the main mechanism of ET is the quenching of the excited singlet state of ^1^Trp* by KP in the ground state. Note that NMR spectra and CIDNP effects of (*R*,*S*) and (*S*,*R*)-configurations of both dyads (I and II) are coincide, as expected for enantiomers.

**Figure 3:**
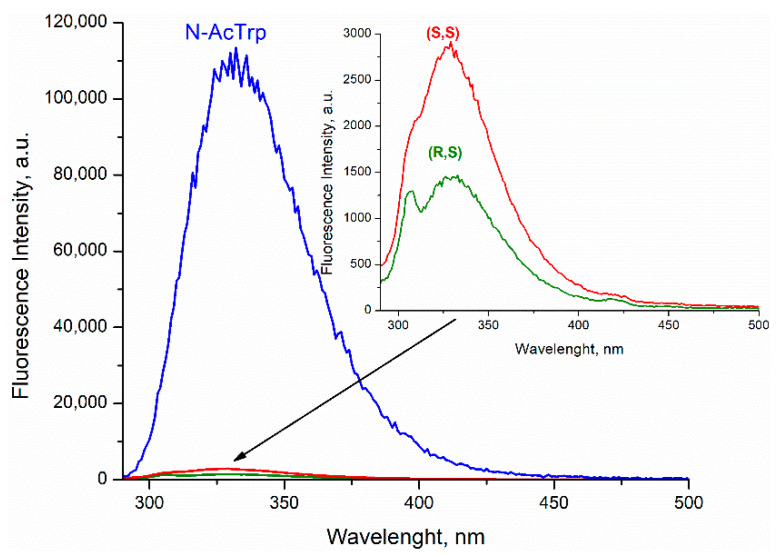
The corrected fluorescence emission spectra of KP-Trp dyad compared to the parent N-acetyl-tryptophan methyl ester in acetonitrile, λ_exc_ = 280 nm.

Comparison of the fluorescence quantum yields of Trp in diastereomers of dyad II with the quantum yield of N-acetyl-tryptophan methyl ester showed extremely effective fluorescence quenching in the dyad (Figure 3).

**Scheme 2:**
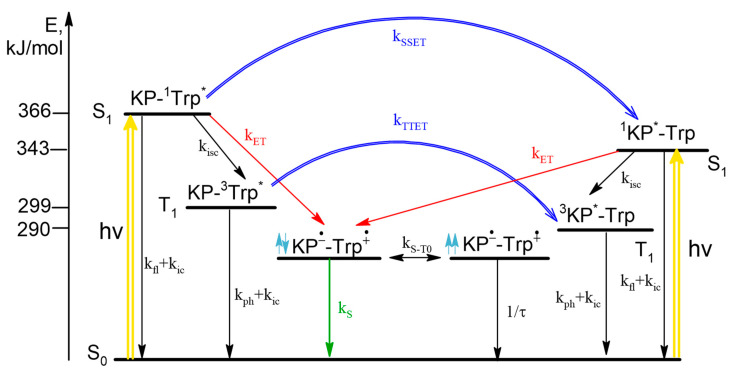
Photoinduced processes in dyad II. The ratio of light absorbed at λexc = 308 nm by KP/Trp is 55/45, at λ_exc_ = 280 nm 65/35. This scheme is similar to the case of dyad I, the difference is that back ET is allowed only from the singlet spin state of BZ. As appears from above, when discussing the CIDNP effects, only the left part of photoinduced processes should be considered.

Such low fluorescence quantum yield was accounted for concurrent SSET and ET (Scheme 2).

In this case, stereoselectivity was observed in fluorescence quantum yields φ_SS_/φ_SR_ = 2 (Figure 3). Despite the report of some differences in the quantum yields of (*R*,*S*)- and (*S*,*R*)-enantiomers [20], the inaccuracy in determining the quantum yields of enantiomers is probably due to the very small amount of free tryptophan in the dyad. Thus, careful analysis of CIDNP effect and fluorescence quenching allowed one to detect stereoselectivity and difference in the CIDNP enhancement coefficients (spin selectivity) of intramolecular electron transfer in diastereomers of dyads with L- and D-tryptophan.

## 3. Peculiarities of Spin Effects in Chiral Linked Systems: Experimental Results

In the previous section, CIDNP was used to prove stereoselectivity of ET in dyads; in this section, we discuss sensitivity of CIDNP and SS to the structure and properties of paramagnetic species with L- and D-Trp. Spin effects, specifically the chemical polarization of nuclei and electrons, have demonstrated a high sensitivity to the structure of diastereomers and their activity in photoinduced ET [14,16]. SS is a recently discovered property of chiral systems that appears in the difference of CIDNP enhancement coefficients (K) of (*R*,*S*)- and (*S*,*S*)-diastereomers under the UV irradiation [14,15]. SS refers to the difference in the K calculated per radical pair, in our case, per biradical-zwitterion (BZ). The K value for each proton in dyad is the ratio of the signal intensity of polarized proton (I_pol_) to the intensity of its equilibrium signal in NMR spectrum (I_eq_). This ratio, so-called “observed CIDNP coefficient”, is divided by the concentration of biradical-zwitterion [BZ] [14]. Thus, SS is the ratio of CIDNP enhancement coefficients for diastereomers of dyads normalized to concentrations of BZ:

(1) $${\mathrm{SS}}_{\left(R,S\right)/\left(S,S\right)}=\frac{I_{\mathrm{pol}}^{\mathrm{RS}}I_{\mathrm{eq}}^{\mathrm{SS}}{\left[\mathrm{BZ}\right]}_{\mathrm{SS}}}{I_{\mathrm{eq}}^{\mathrm{RS}}I_{\mathrm{pol}}^{\mathrm{SS}}{\left[\mathrm{BZ}\right]}_{\mathrm{RS}}}$$

Table 1, Table 2 and Table 3 below show SS values for dyads I-IV. The mechanisms underlying the formation of hyperpolarization in these dyads are described in detail in the previous section of this review.

Authors of [14,15] investigated in detail the possible causes of SS phenomenon and arrived at conclusion that the origin of spin selectivity is difference in HFI constants in the BZ of diastereomers. This is a rather important conclusion, since the difference in the distribution of the spin density also implies a difference in the distribution of the electron density. This, in turn, may be one of the reasons for the difference in the medical activity of drug enantiomers [17]. This is quite probable reason, considering that the diastereomers consisting of enantiomers of drugs linked to chiral amino acids residues can be an analogue of the ligand-receptor or substrate-enzyme complexes [14,15]. In addition, it is important to note that optical isomers of amino acids also show SS (Table 2).

Meanwhile, studies of dyads involving enantiomers of another NSAID—ketoprofen linked with enantiomers of tryptophan (set of (*S*,*S*)-, (*S*,*R*)- and (*R*,*S*)-optical configurations) showed an almost tenfold difference in CIDNP enhancement coefficients (Table 3). 

The reasons for such strong differences will be discussed in detail in the next section. Here we note that the difference by an order of magnitude is hardly related to the difference only in the values of HFI constants. An additional cause may be the difference in the BZ conformations of different optical configurations. This is confirmed by the MD data for diastereomers and by the following peculiarity of their hyperpolarization: the spin density distribution in BZ of chiral linked systems differs from those in a similar radical ion pair. The lack of proportionality between the CIDNP intensity of individual protons and the HFI constants of the corresponding radical ions is illustrated by the data of the Table 1, Table 2 and Table 3.

Thus, this deviation from the fundamental property of high-field CIDNP, i.e., the proportionality between the values of HFI constants and the intensity of polarized NMR signals, also indicates a difference in the distribution of spin density in the BZ of different optical configuration. However, the difference, reaching an order of magnitude for the (*R*,*S*)- and (*S*,*R*)- compared to (*S*,*S*)-diastereomer of dyad II, forces us to suppose that the hyperpolarization of chiral systems has as yet unknown features.

To study the reasons for the anomalously low CIDNP intensity of the (*R*,*S*)- and (*S*,*R*)-diastereomers of dyad II, the authors carried out a modification of the classical radical pair theory by taking into account the dipole-dipole interaction of electrons in the BZ [20](see Section 5). The theoretical section describes the secular equation of spin chemistry including the dipole-dipole interaction of electrons. The result of this modification is the suppression of the singlet-triplet conversion, which, within the framework of the S-T_0_ approximation, leads to the appearance of hyperpolarization. The necessary condition for the manifestation of dipole-dipole interaction of electrons is the magnitude of rotational correlation time of polarized compound in solution which is less than certain parameter (inverse value of the amplitude) of the dipole-dipole interaction of electrons (see Section 5). This condition will be satisfied for large particles, in our case it might be associates of dyad’s diastereomers.

Finally, the peculiarities of spin effects in chiral linked systems involve the significant dependence of hyperpolarization, resulting from photoinduced ET, on a number of conditions: K depends on the diastereomers concentrations, SS depends on the solvent (Table 4) and on the ratio of diastereoisomers concentrations upon UV irradiation of their mixture.

**Figure 4:**
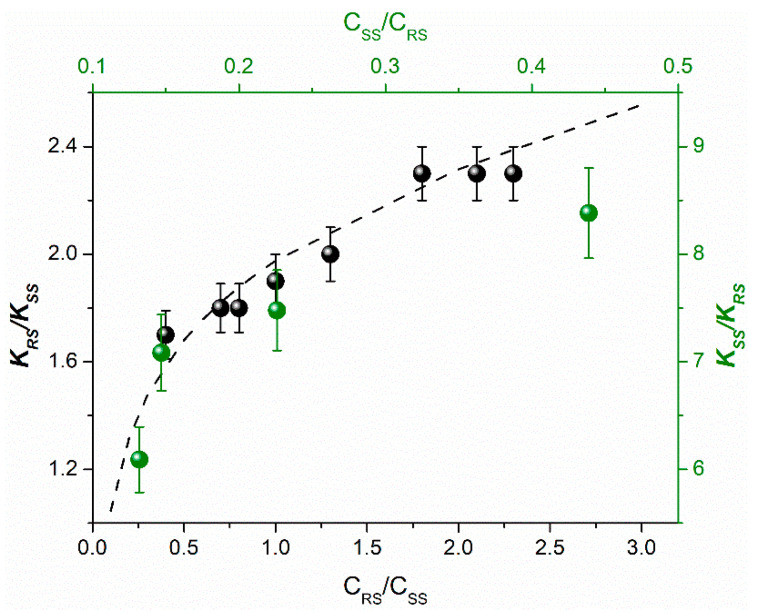
The experimental dependence of the observed ratios of CIDNP enhancement coefficients and calculated values (dash line) on (*R*,*S*)-diastereomer concentration for dyads IV (black balls, C_SS_ = 1 mM) and II (green balls, C_SS_ = 2 mM) [14,20].

The dependence of SS values on the solvent may be accounted by different BZ geometry for diastereomers with different optical configurations in different solvent mixture. Since SS is ratio of the CIDNP enhancement coefficients of diastereomers related to one BZ, the dependence of the SS value on the ratio of diastereomers concentration under UV irradiation of their mixture requires explanation. The explanation has been given in the works [14,15]: the authors associated this dependence with the fact that the process of ET, in which the CIDNP was formed, occurs in a dimer. Dimerization of dyads with naproxen has experimental confirmation [14,15]. The possibility of the dimers formation is also confirmed by the MD data (see Section 4). Below is the comparison of the SS dependences on the ratio of diastereomers concentration measured for dyads I and II (Figure 4).

It should be emphasized, that SS dependences on the ratio of diastereomer concentrations are similar for these two dyads only in the range of high concentrations of (*S*,*R*)-diastereomer of dyad II. The pattern obtained at lower concentrations of the dyad II is presented in Table 5.

An analysis of the changes in hyperpolarization values from Table 5, resulting from the sequential addition of one or another diastereomer to the mixture, leads to the following conclusions. With an increase in the concentration of (*S*,*S*)-diastereomer in the mixture, its hyperpolarization decreases, while hyperpolarization of the (*S*,*R*)-analog, on the contrary, slightly increases when (*S*,*S*)-diastereomer is added. This suggests that, under these conditions, diastereomers form more complex structures than dimers—larger aggregates, that leads to a decrease in hyperpolarization due to the action of the electron dipole-dipole interaction. At the same time, in the case of (*S*,*R*)-diastereomer, the addition of an analog with a different optical configuration reduces the contribution of large particles in favor of smaller associates, leading to increase in hyperpolarization. Thus, the high sensitivity of the CIDNP to the any changes in chiral linked systems allow us to notice that a change in the optical configuration of even one dyad partner leads to noticeable changes in the structure of the associates. The analysis of MD data leads to the same conclusions that the probability of dimerization of the (*S*,*S*)-diastereomers for both dyads I and II less than for the (*R*,*S*)-analogs. Furthermore, the (*S*,*S*)-configuration demonstrates higher stability than (*R*,*S*)-analog, according to the data of quantum-chemical simulation of dyad II diastereomers [20] (see following section).

In conclusion of this part, it should be noted that using the polarization of electrons that directly registers the paramagnetic species, BZ was also detected and the difference between (*S*,*S*)- and (*S*,*R*)-diastereomers of dyad II was shown [16].

## 4. Molecular Dynamics and Quantum-Chemical Simulations of Dyads I and II

This section briefly describes conformational exploration of dyad’s diastereomers by molecular dynamics and quantum-chemical simulations. The results of MD have shown several differences between optical isomers of dyads with L- and D-Trp residues [20]. Such conformational parameters as the values of mean angles between planes of donor and acceptor and contact time of donor and acceptor moiety’s turned out to be different for (*S*,*S*)-, (*S*,*R*)- and (*R*,*S*)-isomers (Table 6).

**Figure 5:**
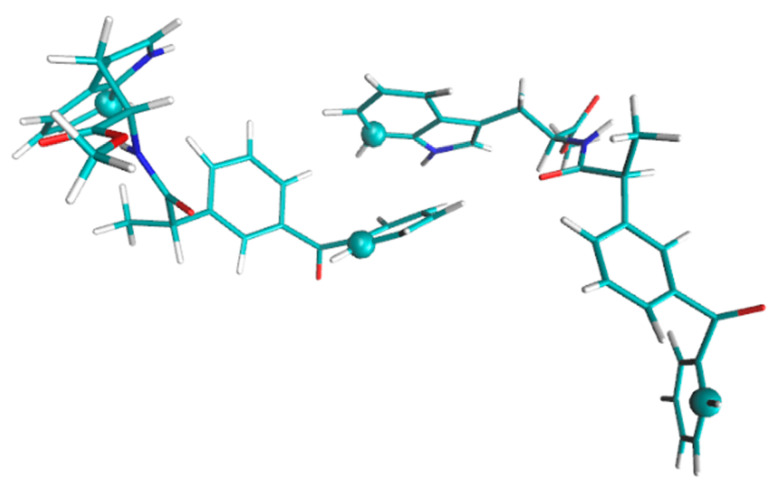
The approach of two molecules of (*R*,*S*)-diastereomer of dyad II, forming a dimer. The distance between atoms marked by spheres was analyzed [20].

**Figure 6:**
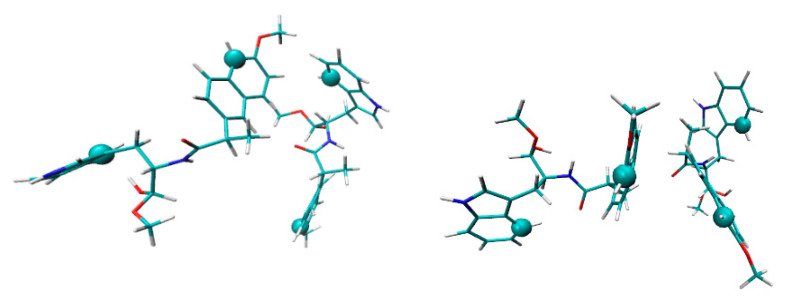
The approach of two molecules of (*S*,*S*)-diastereomer of dyad I, forming a different types of dimers: approach of a NPX fragment with Trp (left) and two NPX residues (right). The distance between atoms marked by spheres was analyzed [20].

An attempt has also been made to assess the probability of dimers formations for various optical configurations of dyads. For dyad I, collision complex were formed either by approaching naproxen residues or by approaching a NPX residue and a Trp residue. In the case of dyad II, the closest approach was observed for KP and Trp residues (Figure 5 and Figure 6). Results of dimers simulations are presented in Table 7.

**Figure 7:**
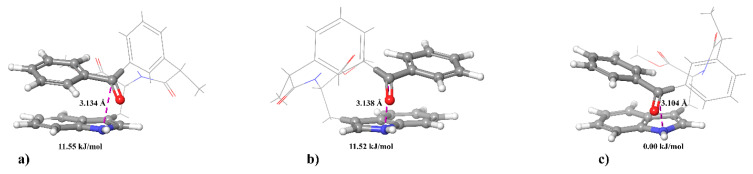
The optimized structures of (**a**) (*R*,*S*)-; (**b**) (*S*,*R*)-; (**c**) (*S*,*S*)-diastereomers of dyad II. Values of Gibbs energy are given relative to (*S*,*S*)-diastereomer [20].

The results of quantum chemical calculations for dyad II have shown no significant differences in the distances between the nitrogen atom of Trp and the carbon atom (C=O) of KP (Figure 7). At that, (*S*,*S*)-diastereomer has the lowest energy, while (*R*,*S*)- and (*S*,*S*)- have the almost equal energy values.

## 5. Theoretical Description of Spin Effects in Chiral Systems: Modification of Radical Pair Theory including Dipole-Dipole Interaction of Electrons

In elucidating the possible reasons of abovementioned peculiarities of the CIDNP effects in chiral linked systems, we theoretically studied the influence of such a factor as the magnetic dipole–dipole interaction of electrons in various optical configurations of diastereomers on the CIDNP efficiency. In [21], in order to clarify the fundamental possibility of such an influence, we considered the simplest static model that does not explicitly include the molecular motions of the reagents. The formation of hyperpolarization was believed to occur according to the traditional mechanism of radical pairs. The CIDNP is determined by the intensity of quantum transitions between singlet S and triplet T_0_ spin states under the action of various spin interactions. These are the Zeeman interaction of dyad electrons with an external strong magnetic field, HFI and magnetic dipole-dipole interaction of electrons. For simplicity, a model was considered with the following approximations: each electron interacts with one magnetic nucleus and the HFI constants (a_1_ = a_2_) are the same for both BZ partners, and the Zeeman interaction of nuclei with an external magnetic field was not taken into account. Based on the solution of the system of equations for the stationary elements of the density matrix, it was found that the magnetic dipole-dipole interaction, as well as the difference between the Larmor frequencies and the HFI, leads to an additional splitting of the S and T_0_ levels of the spin states (which reduces the intensity of the singlet-triplet transitions) and, accordingly, to a decrease in the stationary polarization with an increase in the magnitude of the magnetic dipole-dipole interaction. The magnitude of the magnetic dipole-dipole interaction is known to depend on such parameters as the distance and effective orientation of the radius vector between moieties of dyad (where unpaired electrons are delocalized) relative to the external magnetic field. It is important that for physically justified values of these parameters, the splitting of spin levels due to the HFI and the magnetic dipole–dipole interaction is approximately the same, and the magnetic dipole–dipole interaction can significantly affect the CIDNP value. The possible existence of a difference in these parameters for (*S*,*S*)- and (*R*,*S*)-configurations will lead to differences in the hyperpolarization values. Using the data of quantum chemical calculations for the geometry of the (*S*,*S*)- and (*R*,*S*)-configurations, it was concluded [21] that it is possible to obtain the contributions of spin selectivity to the CIDNP value from **K**_(*S*,*S*)/(*R*,*S*)_ ≈ 0.9 to **K**_(*S*,*S*)/(*R*,*S*)_ ≈ 7.

However, it should be kept in mind that the motion of the reagents can significantly affect the magnitude of the magnetic dipole-dipole interaction. The simplest form of such a motion is the transition between two positions of reacting centers in a dyad that was studied within the two-position model. Within the framework of this model, one position corresponds to a close arrangement of centers, when recombination due to electron transfer is significant and there is a strong magnetic dipole–dipole interaction. In the second position, the centers are far enough away that recombination does not take place, but the dipole-dipole interaction is preserved, although it is weaker. An analysis of the model based, as before, on the consideration of the system of equations for the stationary elements of the density matrix, showed that at different distances in the reaction zone (in the first position) (r_A1_ = 3 Å and 4 Å), hyperpolarization is completely suppressed for any residence times used systems in the reaction zone.

Another significant molecular motion capable of averaging the magnetic dipole-dipole interaction and thereby increasing the polarization is the rotation of BZ as a whole (i.e., the rotation of the radius vector between the nuclei relative to the direction of the external magnetic field). Complete averaging of the magnetic dipole-dipole interaction of electrons occurs if the parameter ξ of the rotation rate that is the product of the amplitude of the magnetic dipole-dipole interaction and the rotational correlation time (τ_c_), is much less than 1. An estimate of the corresponding characteristic correlation time gives a value of 0.16 ns, and the amplitude magnetic dipole-dipole interaction (at r_a_ = 3 Å) is 6.068 × 10^9^ s^−1^ [20]. The parameter ξ in this case is quite large and complete averaging does not occur for such values of the physical parameters. To estimate the dependence of the characteristic time of rotational orientation on the particle size, the well-known expression was used:

(2) $$\tau_{c}=\frac{1}{2D}=\frac{3\mathrm{hV}}{k_{B}T}=\frac{4{\pi \mathrm{hR}}^{3}}{k_{B}T}$$

where τ_c_ is the characteristic rotational correlation time, D is the rotational diffusion coefficient, h is the viscosity of acetonitrile (0.35 × 10^−3^ Pa×s). Thus, the dipole-dipole interaction is not averaged if the particle size is much larger than 5.5 Å. Other mechanisms are also possible, leading to different rates of rotational relaxation and, consequently, different degrees of averaging of the magnetic dipole-dipole interaction for (*S*,*S*)- and (*R*,*S*)- and (*S*,*R*)-configurations [20]. An unambiguous solution to the problem of averaging this interaction requires experimental studies of the rotational mobility.

## 6. Chiral Inversion (CI) in Model Systems and the Possible CI Roles in Alzheimer’s Disease

As mentioned in the introduction, the nature of neurodegenerative diseases, including Alzheimer’s disease, is largely related to amyloid Aβ42 [1,2,3,4]. At the same time, research of this amyloid is carried out in several directions, on the one hand, the toxicity of oligomers of this amyloid relative to brain cells is studied [5,6,7,8,9,10,11], and on the other hand, its structure is explored [3,4,9]. One of the main questions: how does the structure of this peptide change when of L-amino acids are replaced by D-analog? Since classical high-resolution NMR and X-ray methods are inapplicable in the study of highly disordered aggregated proteins and peptides, studies of model systems seem to be promising for determining the role of D-amino acids [3]. In the previous sections of this review, we discussed the effect of L-amino acid replacement to D-analog on the structures of dyad’s diastereomers and the efficacy of tryptophan fluorescence quenching through the photoinduced ET and energy transfer.

Another important question concerns the mechanism of such a replacement—spontaneous chiral inversion occurring in proteins and peptides of elderly patients with neurodegenerative diseases [2,4]. The works carried out in this direction have not yet led to the establishment of the CI molecular mechanisms, but have already demonstrated the significance of this process for the amyloids toxicity [22,23,24,25].

Thus, the use of multidimensional ion mobility-mass spectrometry (IM-MS) and the kinetics measurement of the chiral transformations of Aβ42 amyloid and its fragments in solution provided structural and molecular basis for analyzing the roles of the D-amino acid residues in the structures and properties of amyloid oligomers and fibrils. Today mainly spontaneous CI and joint isomerization of residues of two amino acids, aspartic acid (Asp) and serine (Ser), have been studied. The joint isomerization of Asp and Ser results in aggregation of the long Aβ N-terminal N-segment responsible for penetration into the membrane, where it forms new calcium channels. At the same time, individual inversion of the amino acid residues of Asp and Ser leads to the formation of aggregate of the short C-terminal fragments, which caused inflammation of the microglia [23].

As for the phenomenon of CI itself, since the last century it has been known that amino acids and peptides can undergo chiral inversion when the temperature rises to 100 degrees Celsius, during recrystallization, evaporation and transition from one state to another [1,26].

As regards the molecular mechanism of CI, the computational works, as a rule, suggest an ionic mechanism (see, for example, CI of 2-aminopropionitrile [27]). Numerous examples of CI in NSAIDs have been described in literature, analysis of these experimental data also allows one to suggest that CI occurs through ion intermediates [28]. One of such examples is the CI of (*R*)-enantiomers of NSAIDs proceeds through the enzyme-catalyzed formation of an “activated” derivative of coenzyme A, followed by epimerization to form the (*S*)-enantiomer and, finally, hydrolysis (*S*)-acyl-coenzyme A. Inversion is feasible because after enzymatic deprotonation, an enol-type intermediate is formed, then this intermediate can be reprotonated by enzyme from either side of the planar double bond.

**Figure 8:**
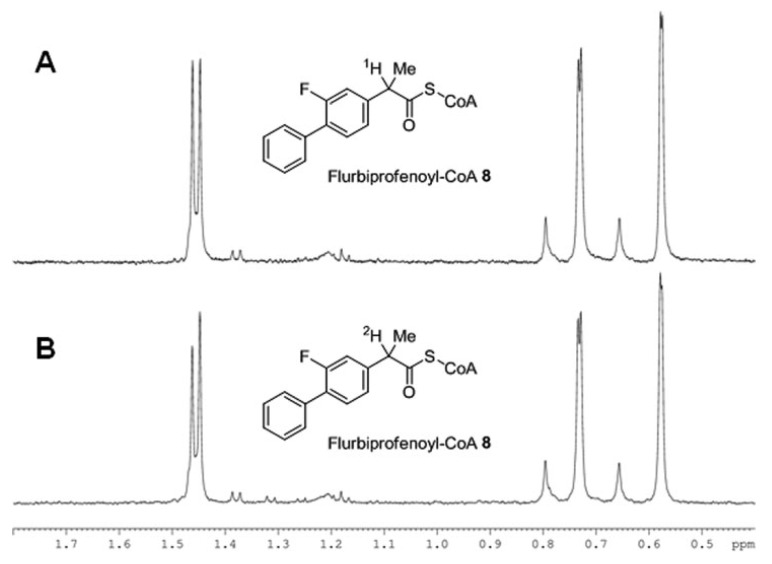
^2^H-Exchange of ±-flurbiprofenoyl-CoA ester under the action of AMACR that shows change in methyl group multiplet at d 1.45 ppm. (**A**) Heat-inactivated enzyme; (**B**) Active enzyme. Reproduced from [29] with permission from The Royal Society of Chemistry.

Second example, confirming the above mechanism, concerns the CI in NSAID (*R*)-flurbiprofen occurring under the action of the α-methylacyl-CoA racemase (AMACR) [29]. In this case (*R*)-enantiomer undergoes a unidirectional transformation to the (*S*)-analog as a result of hydrolysis of its coenzyme A ester. The authors prove the act of the chiral center deprotonation through the formation of the enol form using the deuterium exchange reaction. NMR spectra in the Figure 8 demonstrate change in the ratio of the doublet components of the methyl group located in the chiral center due to change of the α-proton to deuterium.

However, there are also examples in the literature that examine the participation of free radicals, ion-radicals and biradicals in CI [30,31,32,33,34,35].

**Figure 9:**
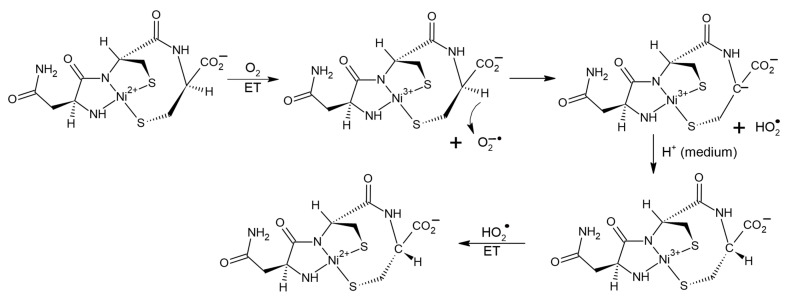
Proposed mechanism of chiral inversion in the Ni^2+^-asparagine-cysteine-cysteine metallopeptide.

New information about the factors governing CI can be gleaned from studies of metallopeptides, considered as close analogues of metalloproteins [30,31,32,33,34]. The effect of the transition metals Co (II), Co (III), Cu (II), and Ni (II) on the racemization of chiral systems was established long ago [30]. Currently, metallopeptides are positioned as devices for biotechnologies, such as drug delivery vehicles, substance for increased site-specific modification of proteins, etc., as well as models for the study of metal-containing enzymes. So, Ni-asparagine-cysteine-cysteine is considered an analogue of superoxide dismutase and it is often used to simulate elementary biological reactions [31]. It has been shown that the binding of peptides to transition metals noticeably increases its reactivity. In the work [31], in particular, catalysis of CI in the Ni^2+^-asparagine-cysteine-cysteine metallopeptide (NiAsCC) by molecular oxygen was studied. Authors of [31], using a set of physicochemical methods, including CD, electron absorption spectroscopy, ESR, and chemical scavenging have detected the formation of several short-lived intermediates, such as a carbanion with a pro-chiral carbon atom, superoxide, the O_2_^−•^ anion, two more carbanions, and traces of the complex with Ni^3+^. In this case, the CI product is the D-isomer of the Ni^2+^ containing peptide. This allows authors of this review to assume the participation of radical steps in CI of Ni-asparagine-cysteine-cysteine complex. The possible scenario might be the following. The catalyzer—oxygen oxidizes Ni^2+^ to Ni^3+^ state, simultaneously turning into O_2_^−•^. The latter withdraws alpha proton from the chiral center in NiAsCC leading to the formation of the HO_2_^•^ radical and a pro-chiral carbanion. During the back attachment of the proton from the medium to the carbanion optical configuration of NiAsCC changes (CI). Then Ni^3+^ is reduced to Ni^2+^ under the action of HO_2_^•^ radical (Figure 9).

The participation of the superoxide anion in the CI of the metallopeptide proved in the work [31] is of interest for this review in two aspects. First, transition metals present in the body, in particular Cu^2+^ and Fe^2+^, are known to act on the aggregates of amyloid Aβ42, leading to its disaggregation [9]. Transition metals are even used to treat Alzheimer’s [9,22]. In addition, it is reasonable to assume that one-electron transfer may be involved in CI process in peptides during the interaction with transition metals. The second aspect concerns the known role of oxidative stress in neurodegenerative diseases [6,7,8]. It is now clear that reactive oxygen species are capable of initiating CI in enzymes and peptides. On the other hand, Aβ amyloids are known to induce oxidative stress in astrocytes and neurons death through activation of NADPH oxidase [7]. These amyloids appear to be both a source and an object for reactive oxygen species. However, which of these functions is the main one to date has not been established in the medical literature.

**Figure 10:**
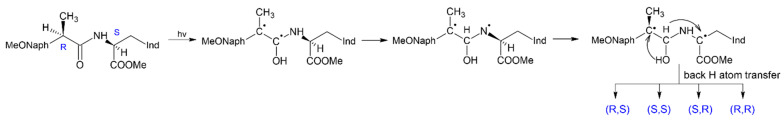
Scheme of photoinduced CI in (*R*,*S*)-configuration of dyad I occurring through the stage of biradical formation and sequential rearrangement in the biradical resulted in formation of a biradical with paramagnetic centers located on pro-chiral centers, where MeONaph is 6-methoxynaphthalen-2-yl, Ind is indol-3-yl, asterisk denotes chiral centers. Adapted from [35], with permission from Elsevier.

The use of photoinduced processes in model donor-acceptor dyads in which reversible electron and proton or hydrogen atom transfer can occur, to study the mechanisms of chiral inversion also seems to be promising [35]. In the case of the coincidence of the paramagnetic and pro-chiral centers in the intermediate formed under UV irradiation, either racemization or inversion can occur in the act of back transfer of a hydrogen atom. This is the case of (*R*,*S*)-configuration of dyad I, in which the mechanism of (*R*,*S*)- into (*S*,*S*)-analog transformation has been established by photo-CIDNP and NMR techniques [35] (Figure 10).

The use of these methods for studying CI became possible because the (*R*,*S*)- and (*S*,*S*)- configuration of this dyad have noticeable differences in chemical shifts and the structure of multiplets of protons located near chiral carbon atoms. Note that photoinduced chiral inversion of amino acids is also described in the literature [1].

So, in this section, cases of chiral inversion in the amyloid peptide, as well as in model systems, are considered. It is noteworthy that the influence of the same factors on CI in real and model systems is traced: reactive oxygen species and metals with variable valence initiate the CI in model systems and affect the aggregation of amyloid peptide.

## 7. Conclusions

Thus, the joint consideration of the results obtained by CIDNP, CIDEP and fluorescence techniques has demonstrated the dependence of the structure of paramagnetic forms of dyads (BZ) and the efficiency of tryptophan fluorescence quenching on the optical configuration of diastereomers. As a result, differences have been shown for dyads containing L- and D-tryptophan. In particular, it was suggested that the differences in BZ structures, reflected in the **SS** values, are related with the formation of associates of dyad’s diastereomers. To explain the dramatic differences between **SS** for dyad with different optical configuration, a theoretical consideration of the possibility of the participation of the dipole-dipole interaction of electrons in the formation of CIDNP has been provided. In addition, analysis of molecular modeling data indicates differences in the optimized conformations and stability of dyads with L- and D-tryptophan residues. In addition, finally, general factors governing the chiral L-D inversion of amino acids in model systems and Aβ42 amyloids containing L- and D-tryptophan have been established. These results, the greater stability of (*S*,*S*)-homo configurations, and the greater tendency of (*R*,*S*)-, (*S*,*R*)-hetero analogs to associate, are consistent with the consequences of the presence of D-amino acids in Aβ amyloids (hetero configuration), namely, the formation of oligomers.

## Figures and Tables

**Table 1:** SS values—the ratio of CIDNP coefficients (K) of protons (marked with red circles) for (*R*,*S*)- and (*S*,*S*)-diastereomers of dyads III (top) and IV (below), and corresponding HFI constants (in G) in BZ [20].

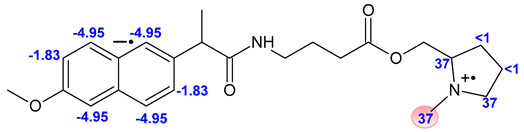	
%ACN	SS_RS/SS_ (N-CH_3_)
100	1.3 ± 0.1
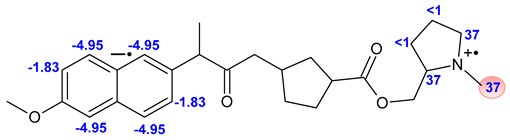	
%ACN	SS_RS/SS_ (N-CH_3_)
100	1.5 ± 0.2
80	2.1 ± 0.2
60	1.9 ± 0.2

**Table 2:** The ratios of HFI constants and observed CIDNP coefficients (K) relative to 1-NH and the values of observed CIDNP coefficients for protons of diastereomers of dyad I in CD_3_CN [20].

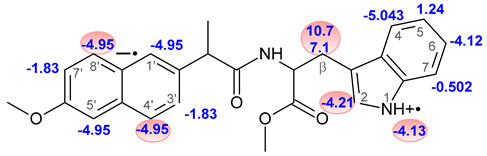				
Position	8′/4′	β-CH_2_	2	1
HFI_i_/HFI_(1)_	1.2	2.6–1.7	1.02	1
K_i_/K_(1)_	1.4–1.1	0.27–0.24	0.064–0.021	1
K_RS_	1.4 ± 0.2	0.27 ± 0.03	0.064 ± 0.07	1.0 ± 0.1
K_SR_	1.4 ±0.2	0.31 ± 0.03	0.025 ± 0.006	1.14 ± 0.07
K_SS_	0.76 ± 0.02	0.16 ± 0.02	0.038 ± 0.003	0.68 ± 0.01
SS_RS/SS_	1.8 ± 0.2	1.7 ± 0.2	1.7 ± 0.2	1.5 ± 0.2

**Table 3:** The ratios of HFI constants and observed CIDNP coefficients (K) relative to 1-NH and the values of observed CIDNP coefficients for protons of dyad II diastereomers in CD_3_CN [20].

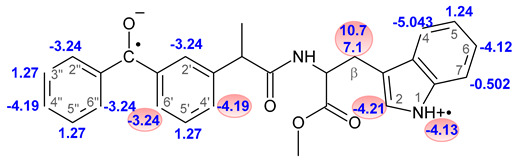				
Position	4′/6′	β-CH_2_	2	1
HFI_i_/HFI_(1)_	0.90	2.6–1.7	1.02	1
K_i_/K_(1)_	0.6–0.5	1.4–1.3	0.3–0.1	1
K_SS_	1.4 ± 0.1	3.0 ± 0.3	0.6 ± 0.1	2.2 ± 0.2
K_SR_	0.11 ± 0.01	0.3 ± 0.1	0.03 ± 0.01	0.21 ± 0.02
SS_SS/SR_	13 ± 1	10 ± 1	20 ± 2	10 ± 1

**Table 4:** SS values measured for protons of dyad I in different mixtures of CD_3_CN/C_6_D_6_ [20].

Volume Fraction of CD_3_CN, %	Ratio of CIDNP Coefficients (SS _RS/SS_) of Diastereomers for Different Protons			
	8′/4′	β-CH_2_	2	1
100	1.8 ± 0.2	1.7 ± 0.2	1.7 ± 0.2	1.5 ± 0.2
80	1.9 ± 0.2	2.2 ± 0.2	1.3 ± 0.1	-
60	1.9 ± 0.2	2.0 ± 0.2	0.9 ± 0.1	-
40	2.0 ± 0.2	1.7 ± 0.2	1.0 ± 0.1	1.8 ± 0.2
20	1.6 ± 0.2	1.8 ± 0.2	1.1 ± 0.1	1.6 ± 0.2
0	1.5 ± 0.2	-	-	2.0 ± 0.2

**Table 5:** SS dependence on the concentration of (*R*,*S*)- and (*S*,*S*)-diastereomers of dyad II [20].

	C_SS_, mM	K_SS_	C_RS_, mM	K_RS_
Addition of (*S*,*R*)- into the (*S*,*S*) + (*R*,*S*) mixture	1.2	4.00	3.6	0.64
	1.2	3.20	5.8	0.43
	1.3	2.70	9.2	0.31
Addition of (*S*,*S*)- into the (*S*,*S*) + (*R*,*S*) mixture	0.9	2.16	5.0	0.23
	3.5	2.24	5.2	0.28
	5.7	1.95	5.4	0.25

**Table 6:** The values of contact times of donor and acceptor moieties at the distances less than 0.6 and 0.45 nm for (*R*,*S*)-, (*S*,*R*)- and (*S*,*S*)-configuration of dyads I-II and angles between plane of donor and acceptor in the solution of acetonitrile (mean values from 10 trajectories with the duration of 100 ns) [20].

Structures of Dyads with Marked Atoms for Distance Calculation (Red) and Planes for Angle Calculation (Blue Circles).	Optical Configuration	Time (<0.6nm)	Angle (<0.6nm)	Time (<0.45nm)	Angle (<0.45nm)
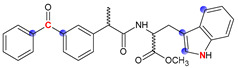	(*R*,*S*)	4.4 ± 0.6 ns	91° ± 4°	1 ± 0.1 ns	81° ± 2°
	(*S*,*R*)	7 ± 0.4 ns	79° ± 5°	0.8 ± 0.1 ns	89° ± 1°
	(*S*,*S*)	7.4 ± 0.3 ns	92° ± 1°	1.5 ± 0.1 ns	105° ± 2°
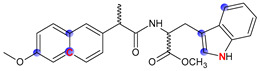	(*R*,*S*)	35 ± 2 ns	77.5° ± 1°	12.3 ± 0.8 ns	95° ± 1°
	(*S*,*S*)	12 ± 1.5 ns	77.5° ± 1.5°	5.1 ± 0.5 ns	89° ± 1.5°

**Table 7:** Probability of dimer formation for various optical configurations of dyads I and II [20].

	The Fraction of Time Spent in Dimer Form, %
(*R*,*S*)-I	0.81 ± 0.08
(*S*,*S*)-I	0.46 ± 0.05
(*R*,*S*)-II	0.6 ± 0.03
(*S*,*S*)-II	0.38 ± 0.04
